# Something’s afoot: podiatry assistants

**DOI:** 10.1186/1757-1146-4-S1-P48

**Published:** 2011-05-20

**Authors:** Angela Reilly, Jenny Elliott, Gaye Witney

**Affiliations:** 1 AHA Podiatry Teacher, Kangan Institute, Private Bag 299, Somerton. Victoria 3062, Australia; 2 AHA Course Coordinator, Kangan Institute, Private Bag 299, Somerton. Victoria 3062, Australia; 3 Program Coordinator, Centre for Better Living, Kangan Institute, Private Bag 299, Somerton. Victoria 3062, Australia

## 

In 2007, Kangan Institute commenced inclusion of Allied Health Assistant Certificate 4 - Podiatry Units delivered by a podiatrist. Different models have been delivered including external placement with private podiatrists, service specific units on site and external work based programs. Since then, a number of TAFE based AHA courses have been commenced including those at Swinburne and Gippsland. With National Registration, the recognised qualification for podiatry assistants is the Certificate III or IV Allied Health Assistant qualification that includes Podiatry Units.

Clarification of the required training for podiatry assistants has offered TAFE services the opportunity to consider the way in which services are offered, and to review the barriers and enablers: (i) to podiatrists using AHA trained podiatry assistants in the work setting and (ii) to acceptance of AHA students for podiatry based clinical placements. This presentation will discuss these issues and present some of the current strategies used in the TAFE sector to enhance the enablers and reduce barriers to the benefit of the profession and its podiatry assistants. It will also discuss methods to expand the training in the future to benefit the podiatry industry.

